# Application of Carbon Nanotubes in Chiral and Achiral Separations of Pharmaceuticals, Biologics and Chemicals

**DOI:** 10.3390/nano7070186

**Published:** 2017-07-18

**Authors:** Ayman L. Hemasa, Nenad Naumovski, William A. Maher, Ashraf Ghanem

**Affiliations:** 1 Chirality Program, Biomedical Science, University of Canberra, Bruce, Australian Capital Territory (ACT) 2617, Australia; Ayman.Hemasa@canberra.edu.au; 2 Collaborative Research in Bioactives and Biomarkers Group (CRIBB), University of Canberra, Bruce, Australian Capital Territory (ACT) 2617, Australia; Nenad.Naumovski@canberra.edu.au; 3 Ecochemistry Laboratory, Institute for Applied Ecology, University of Canberra, Bruce, Australian Capital Territory (ACT) 2617, Australia; Bill.Maher@canberra.edu.au

**Keywords:** carbon nanotubes, chiral separation, achiral separation, single-walled carbon nanotubes, multi-walled carbon nanotubes

## Abstract

Carbon nanotubes (CNTs) possess unique mechanical, physical, electrical and absorbability properties coupled with their nanometer dimensional scale that renders them extremely valuable for applications in many fields including nanotechnology and chromatographic separation. The aim of this review is to provide an updated overview about the applications of CNTs in chiral and achiral separations of pharmaceuticals, biologics and chemicals. Chiral single-walled carbon nanotubes (SWCNTs) and multi-walled carbon nanotubes (MWCNTs) have been directly applied for the enantioseparation of pharmaceuticals and biologicals by using them as stationary or pseudostationary phases in chromatographic separation techniques such as high-performance liquid chromatography (HPLC), capillary electrophoresis (CE) and gas chromatography (GC). Achiral MWCNTs have been used for achiral separations as efficient sorbent objects in solid-phase extraction techniques of biochemicals and drugs. Achiral SWCNTs have been applied in achiral separation of biological samples. Achiral SWCNTs and MWCNTs have been also successfully used to separate achiral mixtures of pharmaceuticals and chemicals. Collectively, functionalized CNTs have been indirectly applied in separation science by enhancing the enantioseparation of different chiral selectors whereas non-functionalized CNTs have shown efficient capabilities for chiral separations by using techniques such as encapsulation or immobilization in polymer monolithic columns.

## 1. Introduction

Carbon nanotubes (CNTs) are allotropes of carbon usually referred to graphite sheets which mainly consist of sp^2^-hybridized carbon atoms. They are wrapped into cylindrical structures and commonly capped by a fullerene-like structure. Once this cylinder is rolled into a single wall, it leads to what is called single-walled carbon nanotubes (SWCNTs). Multi-walled carbon nanotubes (MWCNTs), however, are formed from more than one wall. The structure of carbon nanotube is hybridized as sp^2^ bonding which is even stronger than sp^3^ bonding in diamond. The aspect ratio of CNTs has been found to be more than 10,000,000 which is clearly larger than any other existing material [1]. CNTs have gained enormous attention with more than 100,000 publications by 2015, identifying the importance and their versatility in use due to their unique physical, chemical, electrical and mechanical properties.

The synthesis of SWCNTs requires higher quality control process than MWCNTs and hence, it is more costly to produce large amounts of SWCNTs than MWCNTs. SWCNTs are preferred over MWCNTs when chirality specific structures are needed. Based on chirality, the synthesis of CNTs can be a chirality controlled or a non-chirality controlled process. There are three main techniques used for the synthesis of achiral carbon nanotubes namely arc discharge, laser ablation and chemical vapour decomposition (CVD). Although SWCNTs must be synthesized using a catalyst, MWCNTs can be prepared without a catalyst [2]. Arc discharge is a useful technique to produce both SWCNTs and MWCNTs in large amounts, however, some impurities, undefined chirality, structural defects, random, short and uncontrolled sizes may arise [2]. The main merit of laser ablation method is its ability to produce high amounts of carbon nanotubes with less metallic impurities as compared with the arc discharge technique [3,4]. Unfortunately, this method cannot be applied to large scale production of nanotubes due to the very high costs associated with this process [5]. Currently, catalytic CVD is widely used for the commercial production of CNTs with bulk yield, high purity and easy control of the manufacturing process [6,7]. Thus, the low temperature CVD process has replaced the high temperature arc discharge and laser ablation for the large scale production of CNTs [2]. Controlled synthesis of nanotubes with discrete chirality, however, has not yet been achieved by CVD.

CNTs have been classified into three unique geometries called ‘zig-zag’ (n, 0), ‘armchair’ (n, n) and chiral (n, m) based on how CNTs sheets are wrapped into their tubes [8]. The integers (n, m) represent the number of steps along the carbon bonds within the hexagonal structure. CNTs are considered chiral if n ≠ m [9]. The chirality is believed to originate from the spiral alignment (right- or left-handed) of the hexagonal rings through the axis of CNTs [10]. It has been recently reported that CNTs can be ‘bottom up’ synthesized with definite chirality. This approach depends on two steps namely the synthesis of the macrocycles template structures and the extension of these structures to produce CNTs and relies on the ability to produce CNTs with definite chirality in an efficient, rapid and inexpensive manner [11]. In contrast, CNTs are synthesized as heterogeneous mixtures which impede their applications in the analytical and industrial fields [11]. Therefore, purification techniques are necessary to obtain a homogenous synthesis of CNTs with specific chirality [12,13]. In general, carbon nanotubes can be prepared using one of the following two ways. First, post modification of achiral CNTs is accompanied with chiral selector [14,15,16]. Second, synthesis of chiral CNTs can be performed by the aid of chiral templates [11,15,16]. Essentially, the enantioselective CNTs forms a complex with each enantiomer which then dissociates at different rates from each other leading to the concept of separation. CNTs demonstrated chiral and achiral separation when used as stationary or pseudo-stationary phases in chiral modified chromatographic columns (Capillary Electrophoresis (CE), Gas Chromatography (GC) and High Performance Liquid Chromatography (HPLC)).

CNTs have been used as adsorbent for the preparation of chromatographic stationary phases [15,17,18] and for solid phase extractions [19,20]. It is worthwhile to note that CNTs have been incorporated into polymeric stationary phases to enhance chromatographic separations or attached directly to silica to make stationary phases for the separations of barbiturates and some alkyl benzenes [18]. Derivatization of CNTs has been widely applied in separation science to investigate the ability of CNTs to enhance chiral and achiral separations. It is, however, important, to avoid damaging the pristine structure of CNTs to keep their physical, chemical and chiral properties. For example, strong oxidation reactions of CNTs may alter the physicochemical properties and hinder accurate studies of the possible interactions between the pristine CNTs and analytes [21]. Physical adsorption methods onto silica gel [22,23] seem to preserve the pristine structure of CNTs and thus, they are more suitable than methods relying on oxidation and derivatizations [17]. Many studies refer the recognition capabilities of CNTs to their abilities of making π–π and hydrophobic interactions with analytes [18,24].

MWCNTs were purified with concentrated hydrochloric acid to remove the metallic impurities and maintain the pristine structure of carbon nanotubes. Then, MWCNTs were immobilized by gamma radiation on silica microspheres to preserve the pristine structure of CNTs. Although previous reports explained that the recognition capabilities of CNTs are driven merely by hydrophobic interactions [24], other reports suggest that two or more interactions, such as π–π, dipole-dipole, dispersive forces and molecular sieving, contribute to the adsorption abilities of CNTs [25,26]. It is, however, important to identify if the intermolecular interactions of adsorptions are due to CNTs or other interfering materials. For example, incorporation of CNTs in polymeric stationary phase revealed that polymer played the major role of chromatographic separation which masked the real role of separation by CNTs [26,27]. CNTs have been also shown to play the major role of chromatographic separation due to lowest unoccupied molecular orbital (LUMO) energy [28]. In summary, it is important to keep the pristine structure of CNTs to preserve the exact mechanism of retention by CNTs.

The CNTs with good electrical and sorption characteristics have been widely applied in many fields including chromatographic separation [29]. Some researchers reported establishing efficient, stable and selective techniques by using CNTs for chiral and achiral separation of various compounds [30,31,32]. Progressing CNTs in chiral and achiral separation has been achieved for three main reasons [33]. Firstly, the surface modification of CNTs can be easily performed which renders them chiral stationary or pseudo-stationary phases [33]. Secondly, the CNTs can enhance separation selectivity, column capacity, efficiency and stability of chiral chromatographic separation. Thirdly, CNTs can provide rapid, simple and sensitive recognition of chiral products due to their unique Surface-Plasmon Resonance (SPR) [33]. CNTs have been successfully used as stationary or pseudo-stationary phases in the separation of various compounds via high-performance liquid chromatography (HPLC), gas chromatography (GC) thin layer chromatography (TLC), capillary electrophoresis (CE). The number of articles, covering the application of CNTs as stationary phases in HPLC, is increasing [21,24,26,34,35,36], while the application of CNTs as stationary phase in GC [37,38,39,40] and CE [41,42] to enhance separation of compounds has been recently reported. The information about the application of CNTs in chromatographic separation or solid-phase extraction of compounds is scattered throughout the literature [15,16,20,43,44,45]. There is no review exclusively focusing on the application of CNTs for chiral and achiral separations using HPLC, GC, CE and TLC. The use of non-functionalized CNTs for enantioseparations has been reported with nano-HPLC that utilises immobilization or encapsulation on polymer monolithic columns [46]. Functionalized CNTs with chiral selectors have been reported to enhance enantioseparations with HPLC [47], GC [37] and CE [32,48]. The use of unmodified and functionalized CNTs for achiral separations with HPLC [49] and CE [50] have also been reported. Magnetization of modified or unmodified CNTs has been found to be useful in solid phase extraction of chiral [51] and achiral compounds [52,53]. This review provides the most recent prospects to prepare CNTs with specific properties and hence, the potential use of unmodified or functionalized CNTs for chiral and achiral separation in pharmaceutical, biological and environmental applications.

## 2. MWCNTs for Chiral Separation of Pharmaceuticals

Significant research in the last thirty years has been performed to separate enantiomeric compounds from their racemic mixture using direct chromatographic methods with the aid of highly efficient capillary columns containing chiral stationary phases. The enantioseparation of racemic compounds in an achiral environment is a challenging procedure due to the identical physical and chemical properties of the racemic compounds [33]. Unlike the great success achieved in the field of asymmetric synthesis, the field of chiral separation is still limited due to necessity of using chiral selectors [54]. Up to date, there is no universal chiral selector that can be applied for all chiral separations of all racemic drugs. Chiral separation is theoretically based on fitting the separated enantiomer to a three-point interaction. This theory led many researchers to use different chiral selectors (proteins, cyclodextrin and polysaccharide) as stationary or pseudo-stationary phases in HPLC, TLC and CE through physical or chemical interaction [33].

The MWCNTs with good electrical and sorption characteristics have been widely applied in many fields including chromatographic separation [55]. Although physical studies confirmed the presence of chirality in the structure of carbon nanotubes, the racemic chiral structure of CNTs has not been used alone for enantioseparation [56]. It has been proposed that carbon nanotubes are not able to provide enough energy to differentiate between enantiomers [57,58]. Thus, CNTs were modified with chiral selectors to investigate their ability to enhance the chiral separation of enantiomers. In this section, we discuss and evaluate different approaches for the chiral separation of pharmaceuticals by MWCNTs and SWCNTs (Table 1).

**Figure 1:**
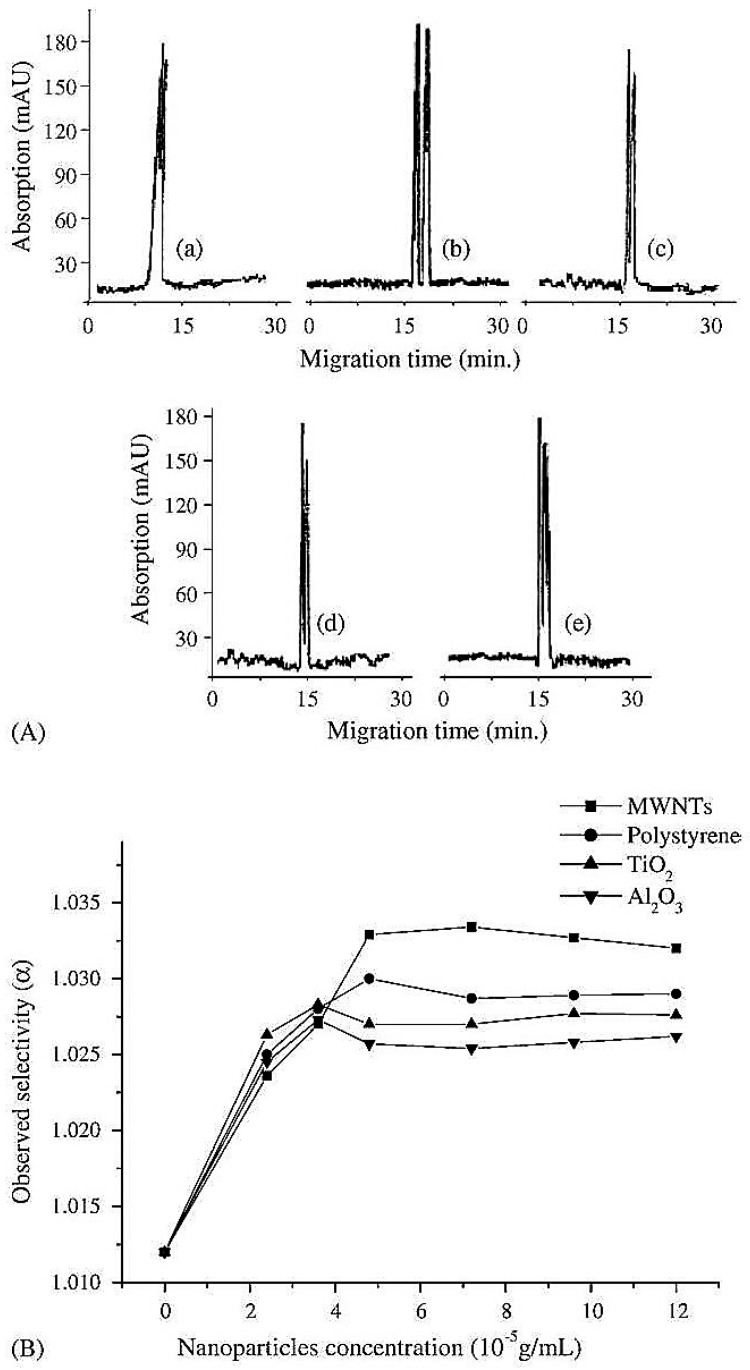
Enantioseparation with CD-MWCNTs “Reproduced with permission from N. Na et al. [48]. Copyright Elsevier, 2006”. (**A**) Different electropherograms showing the effect of CD-MWCNTs on the chiral separation of clenbuterol (**a**) only CD used; (**b**) CD-MWCNTs; (**c**) modified-PS nanoparticles; (**d**) modified-TiO_2_ nanoparticles; (**e**) modified Al_2_O_3_ nanoparticles. (**B**) Change in enantioseparation based on variation in the concentration of the modified nanoparaticles.

The combination of MWCNTs with β-cylodextrin (β-CD) as a pseudo-stationary phase in CE has showed significant enhancement in the enantioseparation of clenbuterol as compared to β-CD only (Figure 1) [48]. In fact, β-CD possesses a unique structure containing a hydrophilic external surface with a hydrophobic inner cavity and has been successfully used as one of the best chiral selectors [61]. It was reported that β-CD can be wrapped helically over the surface of MWCNTs on sonication which led to their solubility in aqueous solvents [62]. This attracted modification of MWCNTs with β-CD to investigate the ability of carbon nanotubes to enhance the chiral separation. The surface of MWCNTs has been modified with β-CD and used as a pseudostationary phase for the separation using capillary electrophoresis [48].

Clenbuterol is a β_2_ agonist that is used in the treatment of respiratory disorders [63]. Furthermore, clenbuterol enhances the retention of the nitrogen and improves muscle growth while reducing body fat and thus, can be potentially abused by some athletes and bodybuilders [64]. Clenbuterol has been marketed as a racemic mixture of S (pharmacologically active form) and R (inactive form) enantiomers. The enantiomers of clenbuterol have been previously separated by different chiral stationary phases [65,66]. MWCNTs-β-CD as a pseudo-stationary phase in CE showed promising enhancement of the chiral separation of clenbuterol. This can be explained by the ability of CNTs to provide large surface area platforms for the interaction between the analyte and the chiral selector β-CD [67,68,69].

**Figure 2:**
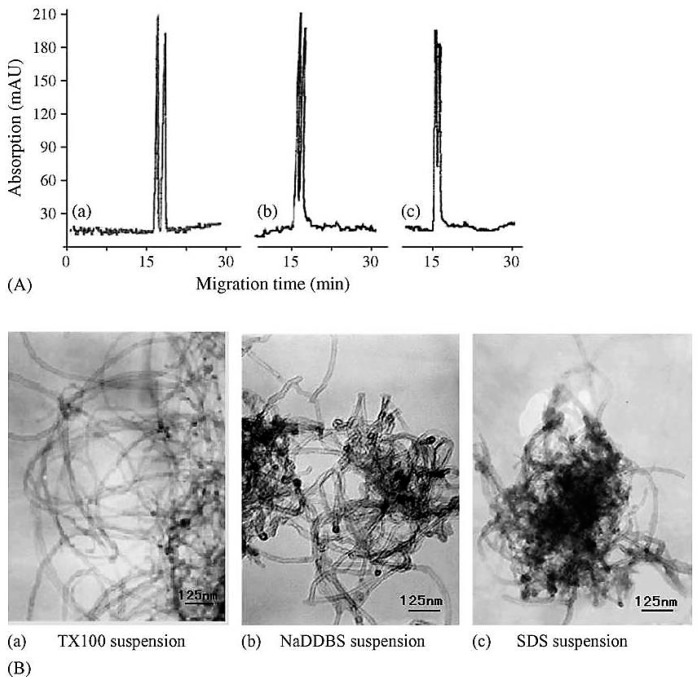
Effect of different kinds of surfactants on the dispersion of CD-MWCNTs and their enantioseparation “Reproduced with permission from N. Na et al. [48]. Copyright Elsevier, 2006”. (**A**) Electropherograms of clenbuterol with CD-MWCNTs dispersed and stabilized in one of the following surfactants; (**B**) Transmission electron microscope (TEM) images of CD-MWCNTs stabilized and dispersed in one of the above mentioned surfactant. (**a**) Trixon X100; (**b**) sodium dodecylbenzene sulfonate and (**c**) sodium dodecyl sulfate.

The poor solubility of the CNTs in most common solvents and the ability to form aggregates due to van der Waals forces is considered to be one of the main challenges for their use [70,71]. Thus, long-chain surfactants have been used to avoid the aggregation of CNTs and to enhance their solubility [72,73,74]. Dispersing the modified MWCNTs into three different surfactants namely; sodium dodecyl sulfate, sodium dodecylbenzene sulfonate and Triton X-100, also showed different capabilities to dissolve carbon nanotubes and hence, the enantioseparation has been impacted [75]. Triton X-100 showed the best dispersing abilities and thus, best enantiosepration of clenbuterol was achieved (Figure 2) [48]. Although β-CD has been also added to the surface of other nanoparticles such as polystyrene, Al_2_O_3_ and TiO_2_, the best separation of clenbuterol was achieved by MWCNTs-β-cylodextrin (Figure 2) [48]. It is the unique structure of CNTs with very large surface areas and its good dispersion in the presence of a surfactant that enhances enantioseparations [49,76]. MWCNTs can also be used to quantify the amount of clenbuterol in urine and blood samples to test for any potential abuse of the use of the drug. In another experiment, the ability of MWCNTs combined with β-CD to enhance the enantioseparation of a racemic mixture of chlorphenirmaine by flow-injection solid phase extraction with fluorescence detection was assessed [77]. The results revealed that the enantioseparation of chlorpheniramine was significantly improved as compared with β-CD without MWCNTs [77]. The following section discusses the merits of the application of MWCNTs in the enantioseparation of clenbuterol for Thin Layer Chromatography (TLC) as compared to CE.

**Figure 3:**
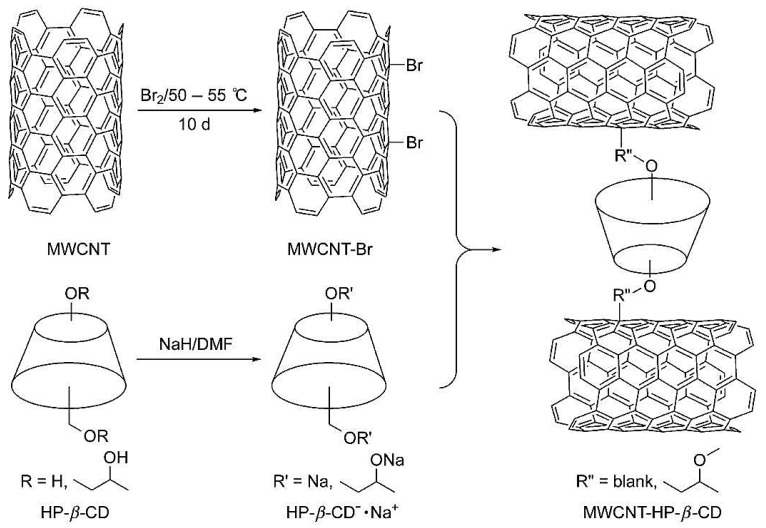
Schematic representation of the preparation of MWCNT-HP-β-CD “Reproduced with permission from Yu et al. [59]. Copyright Wiley, 2011”.

Chiral separation is thus feasible by the modification of MWCNTs with chiral selectors such as β-CD [78]. 2-Hydroxypropyl-β-cylcodextrin (HP-β-CD) was stirred with dimethylformamide (DMF) and sodium hydride for 24 h at room temperature. MWCNTs were brominated [79], and then MWCNT-Br was added to HP-β-CD to initiate a nucleophilic reaction. MWCNT-HP-β-CD was then formed, characterized by FT-IR and investigated as stationary phase in TLC for chiral separation of clenbuterol (Figure 3).

**Figure 4:**
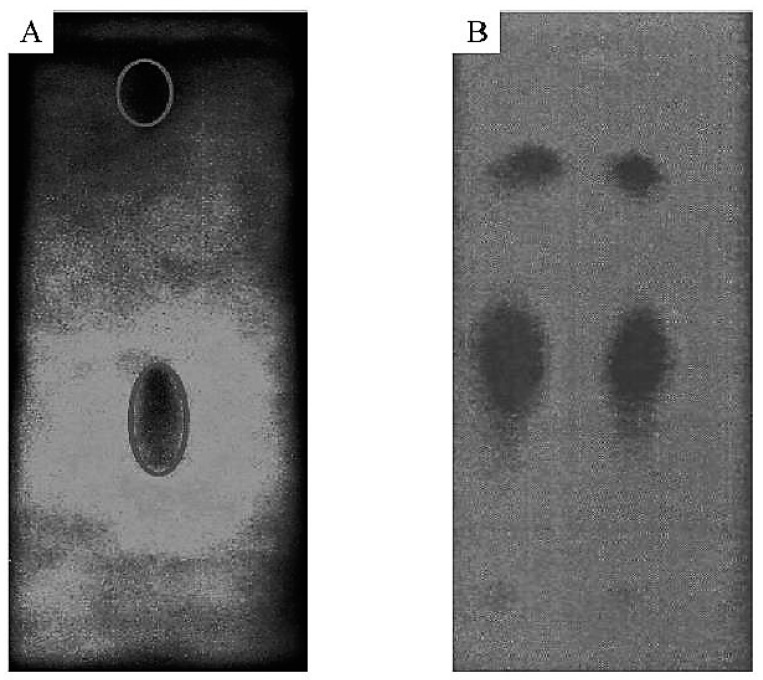
The effect of MWCNTs-HP-β-CD on the resolution of clenbuterol in TLC “Reproduced with permission from Yu et al. [59]. Copyright Wiley, 2011”. Resolution of clenbuterol by using (**A**) MWCNTs-HP-β-CD in TLC (**B**) HP-β-CD without MWCNTs in TLC. This was done at room temerature and with 10 mL of acetonitrile/*t*-butanol (*v*:*v* = 1:1) as a mobile phase.

The functionalization of MWCNTs with HP-β-CD enhances the enantioseparation of metoprolol enantiomers as compared with HP-β-CD without MWCNTs [80]. The application of MWCNT-HP-β-CD for TLC system was found to significantly increase the resolution factor (*R*F = 5.27) of the racemic compound clenbuterol as compared to HP-β-CD only (*R*F = 3.35) (Figure 4) [59]. Although good separation was achieved using HP-β-CD alone, the addition of MWCNT enhanced the enantioseparation dramatically [59]. The resolution factor of clenbuterol in the presence of MWCNT-HP-β-CD has been improved by 57% compared to the use of HP-β-CD only [59]. In this method, TLC was used as it offered several advantages including being cost effective, having high sample throughput, being rapid, easy handled and being efficient chiral separation technique [59]. Excellent enantioseparation of propranolol was also achieved by using c-MWCNTs incorporated into β-CD with TLC [81]. The use of MWCNTs with d-(−)tartaric acid (chiral selector) and silica gel TLC resulted in the resolution of mandelic acid, 2-chloro-mandelic acid and ofloxacin enantiomers [82]. In another TLC-mediated experiment, enantioseparation of ofloxacin was achieved by the use of MWCNTs [83]. Since the use of unmodified MWCNTs or MWCNTs modified with HP-β-CD in TLC showed significant enantioseparation of compounds such as clenbuterol and ofloxacin, CNTs might be useful for the separation of other pharmaceuticals such as fluoroquinolone antibiotics and clenbuterol analogues including β_2_-adrenoreceptor agonists.

MWCNTs have been also combined with a surfactant and used as a pseudo-stationary phase in EKC to enhance the chiral separation of ephedrines. Electrokinetic chromatography (EKC) has been widely applied for enantioseparations having the following advantages, high resolution, efficient separation, cost effective and only requires low quantities of samples and [61,84] reagents. Enantiomeric separation occurs in EKC when a chiral selector such as β-CD is added to a pseudo-stationary phase [85]. Enantiomeric separation has been also studied using chiral surfactants as pseudo-stationary phases in a system called microemulsion EKC (MEEKC) [86,87]. A comprehensive review covering this topic has been recently published [88].

The application of carboxylic-SWCNT has been recently used as a separation carrier in CE [76]. SWCNTs have been recently coated with a surfactant and used as a pseudo-stationary phase in a new EKC system [58]. SWCNTs and MWCNTs were dispersed in a surfactant with the aid of sonication to obtain a homogenous solution that is stable and compatible with EKC. Chiral separations were optimum when CNTs were sonicated for 20 min. An organic modifier was added to micellar EKC to positively affect the resolution by increasing the solubility capacity of the analyte. A co-surfactant, such as 2-butanol, was used in EKC to enhance the permeability of surfactant-coated single-walled carbon nanotubes (SC-SWCNTs) [58]. An organic modifier, such as 2-butanol, was also used to stabilize the baseline and improve the separation. Partial filling technique was applied in this procedure, where the separation solution partially filled the capillary [32]. Thus, a separation zone was produced that is a part of the total separation area. The partial filling technique depends on adding a small amount of concentrated surfactant-carbon nanotubes to enhance the baseline resolution by the interaction between the side wall of CNTs and the racemic compounds [89].

SC-SWCNTs were not able to separate any enantiomer of the tested compounds when applied as a pseudo-stationary phase in EKC [32]. Chirality also exists in the structure of MWCNTs which mainly relies on the way that carbon nanotubes are arranged [32,90]. By applying the partial filling technique to SC-MWCNTs and under optimum conditions of sonication time, pH of the buffer and applied voltage, the enantiomeric mixtures of ephedrine, nor-ephedrine and (1)-*N*-methylephedrine were base-line separated [32]. Ephedrines are classified as central nervous system stimulant drugs and marketed as a racemic mixture of their enantiomers that have different therapeutic and toxicological activities. Several methods such as HPLC [91,92,93], GC [94], TLC [95] have been used to determine the quantity of ephedrine isomers but they suffered from poor accuracy, sensitivity and separation [93]. SC-MWCNTs in EKC demonstrated an inherent ability for the enantioseparation of the tested ephedrines after modification with SDS and sonication for 20 min [32]. The existence of chirality in the structures of SWCNTs and MWCNTs has been confirmed [96]. The use of sonication with a modification of MWCNTs played an important role to reveal the chirality in the structure of MWCNTs and hence, improved their inherent abilities for the enantioseparation in EKC.

**Figure 5:**
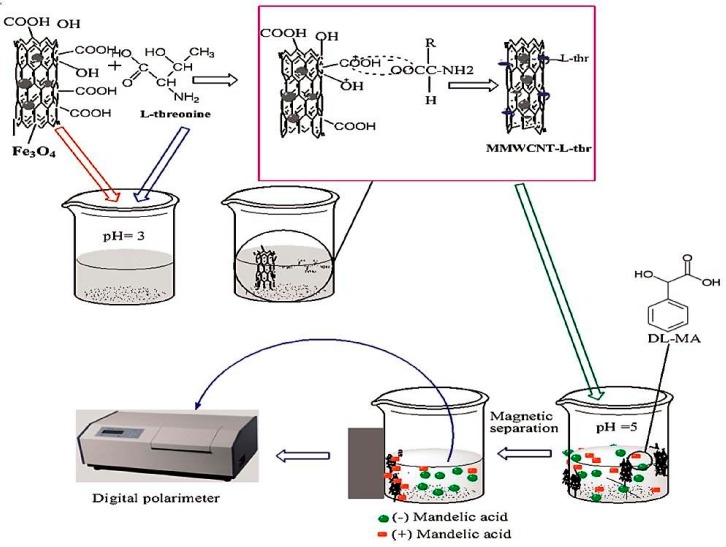
Schematic representation of the enantioseparation of *dl*-mandelic acid with MMWCNTs-*l*-threonine “Reproduced with permission from G.D. Tarigh et al. [51]. Copyright Elsevier, 2015”.

The immobilization of *l*-threonine on MMWCNTs has led to the resolution of the mandelic acid racemic mixture. The R and S enantiomers of mandelic acid are used as chiral analogues in the synthesis of antibiotics such as cephalosporins, penicillin and other pharmaceuticals [97]. Although several methods have been used for the resolution of *dl*-mandelic acid [98,99], they lack high-throughput, simplicity and efficiency [97]. β-CD and CHIRALPAK^®^ IC in HPLC [97], however, showed partial and baseline chiral separation of *dl*-mandelic acid at optimized conditions. Magnetization of CNTs by attaching to iron oxide nanoparticles has been reported as one of the approaches that CNTs adsorb nanostructures to their surfaces [51,100]. Magnetization of CNTs showed efficient partitioning capabilities with reduced cost, time and use of solvents [101,102]. A known chiral selector, *l*-threonine, has been anchored to the surface of magnetic MWCNTs to investigate the ability of MMWCNTs for the enantioseparation of *dl*-mandelic acid (Figure 5). FTIR and X-ray diffraction have been used to confirm the characterization of MMWCNTs-*l*-threonine [51].

*l*-threonine was immobilized on the surface of MMWCNTs through electrostatic and hydrogen bonding [51]. A successful chiral separation of *dl*-mandelic acid was performed by MMWCNTs-*l*-threonine which was then analysed using a digital polarimeter [51]. This showed that strong hydrophobic and hydrogen bonding interactions occurred between MWCNTs-*l*-threonine and the (+)-isomer of mandelic acid more than (−)-isomer. The role of *l*-threonine as a chiral selector was enhanced by its immobilization on the surface of MWCNTs [51]. Thereafter, MMWCNTs have been separated from the solution using a magnetic field in an efficient and quick process. This method offers many advantages over traditional techniques such as being easy, reproducible, inexpensive and reliable [51].

## 3. SWCNTs for Chiral Separation of Pharmaceuticals

**Figure 6:**
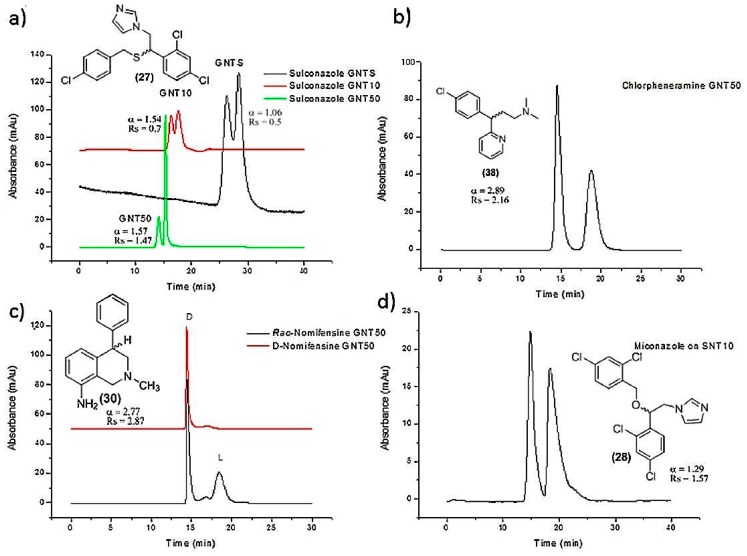
Effect of chiral SWCNTs on the enantioseparation of pharmaceuticals in nano-HPLC “Reproduced with permission from M. Ahmed et al. [46]. Copyright Elsevier, 2014”. Chromatograms obtained for the enantioseparation of (**a**) sulconazole on GMA-columns coated with different SWCNTs concentrations with 45:55 *v*/*v* methanol to water (0.1% TFA) at 240 nm; (**b**) chlorpheniramine on GNT50 with 40:60 *w*/*w* methanol to water (0.1% TFA) at 219 nm; (**c**) nomifensine on GNT50 with 40:60 methanol to water (0.1% TFA) at 219 nm; (**d**) miconazole on SNT10 with 25:75 methanol to water (0.1% trifluroacetic acid (TFA)) at 219 nm. The flow rate was 0.3 μL/min, and all columns were of 150 μm id and 20 cm length.

SWCNTs (6, 5) were modified with carboxylic groups in order to enhance their solubility and selectivity for the separation of small molecules. Thereafter, c-SWCNTs aqueous suspensions were dissolved in two different polymer mixtures to prepare two monolithic columns and investigate the ability of CNTs for chiral separation of pharmaceuticals [46]. The first mixture consisted of ‘20% monomer glycidyl methacrylate, 20% crosslinker ethylene glycol dimethacrylate and 60% porogens (36% 1-propanol, 18% 1,4-butandiol and 6% SWCNTs)’ [46]. The second mixture composed of ‘16.4% monomers (0.4% sulfpropyl methacrylate, 16% butylmethacrylate), 23.6% crosslinker ethylene glycol dimethacrylate and 60% porogens’ the same as the first mixture. [46] The ratio of 40:60 *w*/*w* of monomers to porogens has been selected to provide a monolith with good permeability, reasonable mechanical stability and high surface area [103]. Three different chromatographic modes were used; namely reversed, normal and polar organic mobile phase [46]. The same monolithic columns were prepared in the absence of SWCNTs and used as a reference. Under optimized conditions, the SWCNT monolithic column led to baseline separation of sulconazole, nomifensine, miconazole, celiprolol, etizoline, cizolirtine and chloramphenicol [46]. Partial separation was reported for atenolol, acebutolol, metoprolol, pindolol, tocainide, caprofen and aminoglutithimide. No chiral separation for any of the racemic analytes was observed using the control columns [46]. Successful chiral separations were observed when using reversed phase mode which could be attributed to the high hydrophobic and stacking bonding in the aqueous reversed mobile phase [46]. Baseline separation of nomifensine, chlorpheniramine, was also accomplished in SWCNTs monolithic column using a polar organic mobile phase (2-propanol and methanol) (Figure 6). Furthermore, partial separation of sulconazole (Figure 6), tocainide, *o*-methoxymandelic acid, cizolirtine and glaphenine was observed under the same conditions and using the same column [46]. There was no baseline separation of any racemic compound when a normal mobile phase of n-hexane and 2-propanol was applied. A partial separation was detected for sulconazole and metoprolol upon using normal mobile phase [46]. Generally, the monolithic column prepared with sulfopropyl methacrylate showed stronger retention of the analytes compared with that prepared with glycidyl methacrylate [46]. The columns prepared with SWCNTs with (6, 5) chiral index showed better chiral separation than those with SWCNTs with (7, 6) chiral index. SWCNTs in this case were used without modification of chiral selector, which means that CNTs may possess inherent chiral centers in their structures. This enantioseparation capacity can also be explained by the modification of SWCNTs with carboxylic acid that improves not only their aqueous dispersion but also enhances their enantioseparation. In another experiment, the encapsulation of CNTs in a polymer monolithic column containing glyceryl monomethacrylate (GMM) and ethylene dimethacrylate (EDMA) led to HPLC enantioseparations of a wide range of small molecules [104].The encapsulation of c-SWCNTs in a polymer based monolithic column may also be an appropriate technique for revealing the chirality of CNT.

In another experiment, non-carboxylated and ultra-short SWCNTs have been immobilized by non-covalent process into pre-packed HPLC monolithic columns [22]. CNTs were kept intact to maintain their chirality, the physical and chemical properties of their sp^2^ structure. The results indicated very fast separations of a series of small aromatic compounds as compared with the use of C18 monolithic columns [22]. This method offers an efficient technique for preparing stable HPLC stationary phases with non-functionalized CNTs that can be used for other applications in enantioseparation science.

## 4. SWCNTs and MWCNTs for Chiral Separation in Biological Active Compounds

Chiral analysis of amino acids is necessary in medical, biotechnological and pharmaceutical applications [105,106,107]. Although amino acids exist in nature mainly in the *l*-form, the presence of *d*-form of the amino acids has recently been confirmed in various higher organisms [108]. Thus, it is essential to obtain pure enantiomers of the building blocks for proteins and peptides for their therapeutic or diagnostic applications. Several HPLC methods have been applied with different chiral selectors for chiral separation of the primary, secondary as well as beta and gamma amino acids [107,109,110]. Baseline and partial enantioseparation of some amino acids by modified CNTs have been reported and discussed in the following sections.

The application of SWCNTs and MWCNTs with ionic liquids in GC led to chiral separations of some amino acids. Ionic liquids have unique chemical and physical properties such as low melting points, moisture content and air stability, high solubility and no vapour pressure. Currently, the application of ionic liquids as a stationary phase is of great interest in separation science [111,112,113]. SWCNTs can be bonded to the inner wall of a capillary column and used to assist the addition of chiral selectors. A chiral ionic liquid such as (R)-*N*,*N*,*N*-trimethyl-2-aminobutanol-bis(trifluoromethanesulfon) imidate has been synthesized [114]. Two columns have been prepared, one containing the chiral ionic liquid only (column A) and the other containing SWCNTs bonded to the chiral ionic liquid (column B) [37]. SWCNTs have been added to a chiral ionic liquid in order to investigate the ability of SWCNTs for the enantioseparation of pharmaceuticals [37]. Twelve racemates have been tested and eight of them have been separated by column B in the presence of SWCNTs, whereas only four racemic compounds have been separated by column A in the absence of SWCNTs [37].

In other words, SWCNTs were able to enhance the enantioseparation when coated with a chiral ionic liquid in GC. The enantiomers of (±)-*N*-phenyl-α-methylbenzylamine, *dl*-leucine, (±)-carvone and (±)-*N*,*N*-dimethyl-1-phenylethylamine were separated by column B and not A. In addition, the presence of SWCNTs in the mixture with the chiral ionic liquid enhanced the enantioseparation of (±)-α-methylbenzylamine and (±)-phenylethanol compared with column A without SWCNTs [37]. Column B with SWCNTs showed significant resolution of (±)-phenylethanol compared to column A without SWCNTs [37].

**Figure 7:**
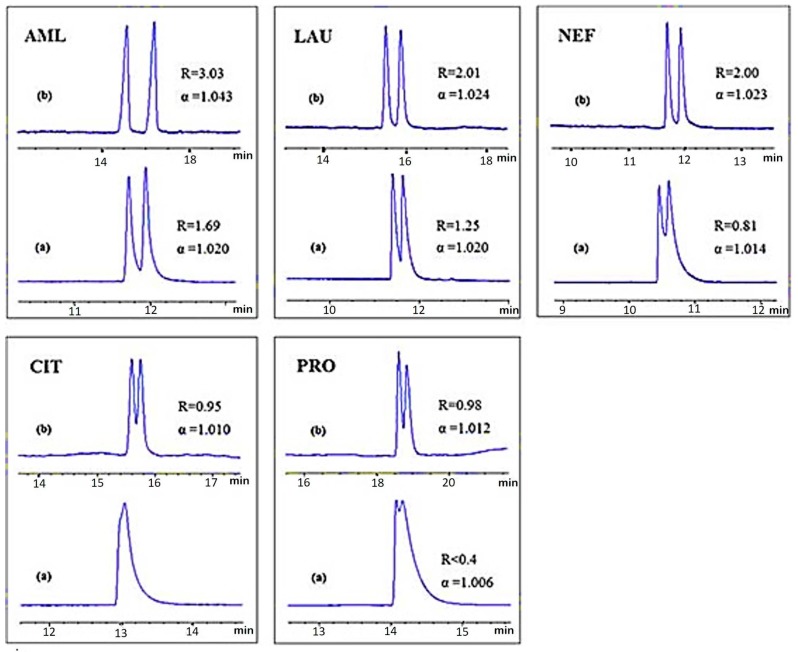
Effect of ionic liquid coated-MWCNTs on the enantioseparation of five drug enantiomers PRO: propranolol, AML: amlodipine, LAU: laudanosine, NEF: nefopam, CIT: citalopram “Reproduced with permission from Q. Zhang et al. [115]. Copyright Wiley, 2014”. (**a**) In the absence of ILs-MWCNTs in the running buffer; (**b**) In the presence of ILs-MWCNTs in the running buffer.

Scanning electron microscope images indicated that SWCNTs are attached end to end and produced a network skeletal-like structure [37]. In another experiment, enantioseparation of citalopram has been reported that utilised an ionic liquid coated MWCNTs for buffer modification in CE as compared with the same system without MWCNTs [115]. A significant enhancement of the enantioseparation of amlopdipine, propranolol, laudanosine, nefopam, sulconazole and ketoconazole has also been reported by using ionic liquid coating carbon nanotubes with a chiral selector (Figure 7) [115,116]. Thus, SWCNTs provide a large inner wall for the interaction between the chiral selector and the analytes [37]. This achievement could be extended to more chromatographic columns and for the separation of a wider range of amino acids and chemical analytes in biological and chemical samples.

**Figure 8:**
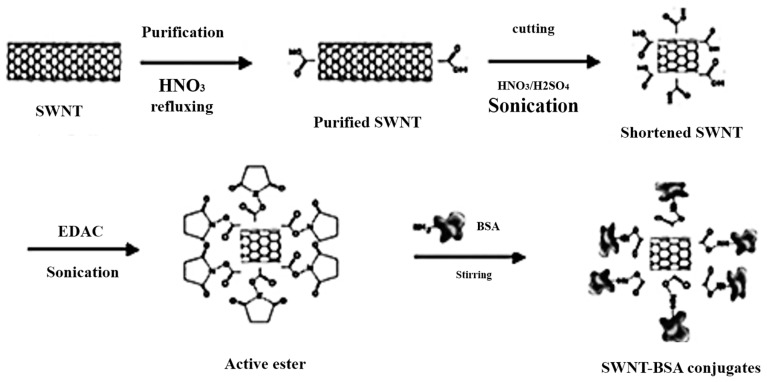
Representative scheme for the preparation of SWCNTs-BSA “Reproduced with permission from Weng et al. [60]. Copyright Wiley, 2007”.

Currently, some proteins such as BSA and HSA have gained attention as chiral selectors for use in capillary electrophoresis [117,118]. More attention has been paid to the biocompatibility of CNTs conjugated with proteins [119]. SWCNTs have been conjugated with BSA to investigate the ability of carbon nanotubes to enhance the enantioseparation of the racemic mixture of tryptophan. SWCNTs were purified from the metallic impurities by reflux in 70% *w*/*w* nitric acid for four hours followed by cutting into short pieces in a chemical oxidation reaction with nitric and sulfuric acid (1:3 *v*/*v*, 70% *w*/*w* and 98% *w*/*w*, respectively) [60]. FT-IR spectroscopy was used for the identification of the carboxylic SWCNTs. Then, the short carboxylic SWCNTs were dispersed in water using ultrasonic agitation [60]. The carboxylic SWCNTs were then attached with BSA via a diimide-activated amidation reaction in the presence of EDAC under ultrasonication [120]. The schematic flow for the production of SWCNTs-BSA is shown in Figure 8.

SWCNTs conjugated BSA was immobilized in a PMMA microchannel for the separation of tryptophan using microchip electrophoresis (MCE) [60]. The stability of this novel protein-SWCNTs-stationary phase was confirmed using microchip electrophoresis MCE [60]. There was no separation of the racemic mixture of tryptophan in the absence of SWCNTs-BSA [60]. Upon adding 0.075–0.1 mg/mL of SWCNT-BSA conjugates, however, baseline separation was observed and then enhanced by increasing the concentration of SWCNT-BSA conjugates. By optimizing separation conditions such as electrical field power, buffer pH and the concentration of SWCNT-BSA conjugates, a successful enantioseparation of tryptophan was achieved in under 70 s with RF of 1.36 [60]. The positive results achieved using SWCNT-BSA conjugates might be extended to the chiral separation of other amino acids indicating the importance of SWCNTsin the biological studies.

The immobilization of SWCNTs with pyrenyl derivative of a chiral aminoglycoside called neomycin A (PNA) has resulted in the enantioseparation of some amino acids. At first, a carbon nanotube monolithic silica based column was prepared. An immobilization of a pyrenyl derivative of a chiral aminoglycoside called neomycin A (PNA) was then performed on the surface of the SWCNTs monolithic column [47]. This PNA-SWCNTs stationary phase was then used to test the ability of SWCNTs for the enantioseparation of ten amino acids. Under optimum conditions, enatioseparation was observed for all of the tested amino acids using PNA-SWCNTs monolithic column [47]. Tryptophan is one of the essential building blocks of proteins and analogues for serotonin and melatonin which improves the mood and sleep [121]. The pharmaceutical use of tryptophan is banned due to the severe side effects [122]. Importantly, the *l*-tryptophan that occurs in food products (within the food matrix) is more applicable [123]. Tryptophan presents as different isomers which induce different pharmacokinetic and pharmacodynamics effects [124]. The separation of the enantiomers of tryptophan has become possible with the aid of some traditional methods such as CE [125], HPLC [126,127], and an electrochemical method [128]. The two enantiomers of tryptophan were partially separated using PNA-SWCNTs as a chiral selector stationary phase [47]. Enantioseparation of tryptophan was, however, more efficient with conventional chromatographic techniques using β-CD as a chiral stationary phase than PNA-SWCNTs. The two enantiomers of *dl*-alanine were completely separated using the same chiral stationary phase [47]. Alanine, also, one of the essential building blocks in the biosynthesis of proteins, where the *l*-form of alanine was found to be one of the essential amino acids in the human genetic code [47], while the *d*-form exists in bacterial cell walls [129].

The best separations were observed when 1 mM of CuSO_4_ was added to the mobile phase. The addition of 1 mM of CuSO_4_ was found to enhance the enantioselectivity of analytes and reduce the analysis time by controlling the dissolution enthalpies and entropies [47]. Amino acids with phenyl rings in their structure such as tryptophan, phenylalanine and tyrosine have long elution times due to an interaction of these phenyl groups with those in SWCNTs and neomycin A [47]. An ionic interaction was proposed between the acidic amino acids and the protonated amino group of PNA [47]. SWCNTs not only offer a hydrophobic site of interaction with benzyl rings of the analytes but also provide a high surface area of interaction between the analytes and the chiral selector PNA [47]. Therefore, SWCNTs modified with a chiral selector can facilitate the chiral separation of amino acids by increasing the area and number of sites of interactions.

## 5. SWCNTs and MWCNTs for Achiral Separation of Pharmaceuticals and Chemicals

The application of SWCNTs and MWCNTs has also been extended to include achiral separation of a mixture of pharmaceuticals; an essential step in quantitative and qualitative analysis [130,131]. Several examples with promising outcomes have been reported where SWCNTs and MWCNTs were tested for their abilities to achieve achiral separations (Table 2) [49,50,52,53,76]. For example, the analysis of catecholamine in blood is important for monitoring the normal and pathological activity of the adrenal gland [132,133]. The HPLC and CE quantification of catecholamine in plasma is a significant marker for some diseases such as pheochromocytoma and hypertension [134,135]. On the other hand, other central nervous system (CNS) stimulants such as caffeine and theobromine existing in tea, cocoa and coffee are also used in respiratory disorders as bronchodilators present in many pharmaceutical preparations. Caffeine usage in particular has been involved in many cardiac, renal, gastric and CNS disorders and classified as a drug of abuse. Therefore, there is a need for the separation of caffeine and theobromine in pharmaceutical, biological and food products. The application of SWCNTs for the separation of catecholamines, caffeine and theobromine is discussed in the following section.

The addition of SWCNTs-modified with carboxylic acids to a run buffer in CE played a significant role in the achiral separation of a mixture containing catecholamines, caffeine and theobromine. It is possible to modify the buffer in capillary electrophoresis to enhance the resolution abilities of the system. It has been indicated that molecular micelles, polymeric phases, can significantly play a role as separation agents in CE. Their polymeric structure not only offers an additional solvation environment but also enhances the stabilization of the mixture by the steric strains due to the covalent stabilization of the polymer mixture [138]. It has been reported that carbon nanotubes added in the buffer system of CE improved the selectivity between different solutes [139]. Since CNTs are insoluble in common solvents, a process of cutting into short pieces was performed followed by chemical oxidation in concentrated acids. This process resulted in carboxylic SWCNTs that were characterized by FT-IR spectrum and then dispersed in a buffer medium. Adding c-SWCNT as additive buffer was applied in CE to investigate the ability of c-SWCNT to improve the electrophoretic behaviour of a solution containing theobromine and caffeine (1:1). The consequences of adding c-SWCNT in the buffer solution were that the migration time of theobromine and caffeine increased, the peak width increased and the peak height was reduced as compared with the buffer solution in the absence of c-SWCNT.

This retention time and the shape of peaks were optimum (Rs = 1.34) when the concentration of c-SWCNTs was 0.1 mg/mL compared to the buffer solution only (Rs = 0.69). Caffeine has an additional methyl group than theobromine that retards its movement resulting in a longer retention time [76]. It is believed that the presence of c-SWCNTs in the buffer solution offers additional interaction sites with the analytes by forming an aggregation like a network with high surface area and molecular sieving properties [76]. The potential molecular sieving properties of carbon nanotubes and graphene has been recently reported [140,141]. These aggregations may occur due to the Van der Waals attractions among isolated CNTs and hydrogen bonding sites of the carboxylic groups attached to c-SWCNTs. Analytes of different pore sizes pass through these aggregates in the separation process which indicates the role of CNTs in the formation of network-like structures and hence the separation of analytes [140,141]. It is believed that the tubule structure of SWCNT also plays a significant role in the separation process in CE [140,141].

The tubule structure of SWCNTs have been totally destroyed by oxidation with concentrated nitric acid [53] and then their performance were compared with intact SWCNTs. Damaged SWCNTs showed no effect on the migration time of caffeine and theobromine whereas; intact c-SWCNTs evidently increased the migration time. The use of SDS buffer alone is effective for the separation when used above its critical micelle concentration. In this experiment, SDS buffer was used at a concentration below its critical micelle concentration to avoid the interference caused by SWCNTs. When SDS buffer was used at concentration above the critical micelle concentration, it separated caffeine and theobromine. The mechanism of action of c-SWCNTs in the separation process, however, has been shown to be independent of the micelle formation with surfactants (1% *w*/*w*) SDS. When SDS 1% (*w*/*w*) (which does not form the micelle network) was used in the buffer medium in the absence of c-SWCNTs, the separation of theobromine and caffeine did not occur [76]. Increasing the migration time due to the network-like structure formed by c-SWCNTs, improves the separation of theobromine and caffeine.

In another experiment, c-SWCNTs was added to the buffer medium in CE to evaluate the separation of homologues compounds namely epinephrine and *dl*-noradrenaline [76]. Acetone was used as a neutral electroosmotic flow marker (EOF). The presence of the multi-charged c-SWCNT in the buffer reduced the electroosmotic mobility by increasing the ionic strength of the buffer and hence, the migration time of acetone increased. An improvement in the shape of the peaks and the migration time of epinephrine and *dl*-noradrenaline has occurred compared to the use of the buffer solution in the absence of c-SWCNT [76]. It has been reported that maximum resolution occurred when the pH was adjusted to be between 8–8.5 [76]. The carboxylic group attached to SWCNTs is dissociated at pH 8–9.5, the dispersibility of SWCNTs is enhanced, the network like structure is easily formed, and thus, the separation capabilities are improved. The addition of c-SWCNTs to the buffer medium not only enhances the resolution but also expands the range of pH which is important in CE due to its high sensitivity to pH changes. In another experiment, c-SWCNTs were added to the buffer medium to investigate the ability of CNTs to separate structural isomers such as catechol and hydroquinone. The results revealed that if c-SWCNT was added to the buffer medium, no increase in the separation of hydroquinone and catechol was observed. An improvement in the peak shape, however, has been reported upon adding c-SWCNTs to the buffer medium, probably because the molecular structures of catechol and hydroquinone are smaller than that of caffeine and theobromine, and thus catechol and hydroquinone cannot be retained by the pores formed by carbon nanotubes. Another explanation is that caffeine and theobromine are more stable in these solutions than catechol and hydroquinone. In other words, catechol and hydroquinone can be oxidized in these solutions and hence unable to react with c-SWCNTs. Another possible reason that the peak shape of the analyte was improved is that the charged c-SWCNT reduces the conductivity between the buffer solution and the analyte. Collectively, c-SWCNTs added into a buffer medium can form an aggregated-like structure that can act as a pseudo-stationary phase for achiral separation of some pharmaceuticals by enhancing the resolution and improving the peak shape [76].

SWCNTs have been incorporated into a silica ionic hybrid stationary phase in HPLC which led to achiral separations of chemicals such as chlorinated herbicides and nucleotides. In fact, SWCNTs have been found to contribute in the electrostatic interactions, dispersion forces, hydrogen bonding, π–π stacking and hydrophobic interactions with analytes [15,16]. An ionic hybrid stationary phase was synthesised by non-covalent immobilization of carboxylated-SWCNTs on amino-derivatized silica gel [22,142]. Then, SWCNTs-ionic hybrid was used to fill an empty column in HPLC [22,142]. The results showed fast and efficient separation of a wide range of aromatic compounds, including benzoic acid derivatives, chlorinated herbicides, nucleotides and Sudan dyes, with good peak asymmetry factor and high theoretical plate number [142]. Sudan dyes, however, contain highly conjugated aromatic structures and hence they form strong π–π interactions with SWCNTs which reduced the theoretical plate number and the peak sharpness [142]. In another experiment, MWCNTs incorporated on silica microspheres [24]. Layer by layer assembly was applied in this method to avoid the strong interactions with aromatic compounds and the long retention times. The results showed improvement in the peak shape and the separation factors as compared with the commercial blank column without MWCNTs [24].

The functionalization of MWCNTs or graphene with polydimethylsiloxane in Micro GC significantly shortened the analysis time and improved the resolution of polar and non-polar hydrocarbon compounds [143]. Micro GC is now widely used in separation science as it is cost and time effective and easy to operate [144,145]. Polydimethylsiloxane is considered to be one of the efficient stationary phases for the separation of hydrocarbons [143]. Graphene is better uniformly distributed on polydimethylsiloxane than MWCNTs do and thus, graphene coated column showed the superior performance for the separation of non-polar compounds [143]. The use of SWCNTs, alone, as a stationary phase in micro GC resulted in the separation of five *n*-alkanes [146]. The use of polydimethylsiloxane in micro GC was useful in the separation of some hydrocarbons [147]. Covalent functionalization of capillary column in GC with graphene alone separated a wide range of organic compounds [148]. The best results, in terms of resolution parameters and retention times, however, have been achieved by combining MWCNTs with Polydimethylsiloxane [143].

## 6. MWCNTs for Achiral Separation in Biologics

The determination of uric and ascorbic acid concentrations in biological fluids such as urine and blood is very important since uric acid is involved in many diseases such as gout [149], Lesch-Nyhan syndrome [150] and type two diabetes [151]. The separations of uric acid from ascorbic acid by voltametric methods with the aid of CNTs have been reported [152,153,154]. Dopamine is a neurotransmitter, and its disturbance, plays a significant role in the pathways of many neurodegenerative and CNS diseases. The separation of dopamine from uric acid with CNTs, using voltametric technique, has been accomplished [155]. Cytosine (C), thymine (T), adenine (A), guanine (G) and uracil (U) are the main blocks in RNA and DNA. If the sequence or structure of DNA or RNA has been changed, protein biosynthesis may be inhibited and thus, many diseases may occur. The separation of purine and pyrimidine bases using CNTs in CZE has been reported [156,157]. The following section discusses different approaches for the application of MWCNTs for achiral separations in biological studies.

MWCNTs were found to enhance the electron transfer reactions if used as an electrode. Owing to their physical, chemical, mechanical properties, high surface area and many functional groups, CNTs can be used to initiate catalytic reactions [136]. A solution of β-CD with CNTs was precipitated on the surface of an electrode to investigate the ability of CNTs to enhance the chemical separation of uric and ascorbic acids [53]. The use of MWCNTs offered high surface conductive area and facilitated the transfer of electrons between the analyte and the electrode. β-CD was immobilized on the surface of the pores of CNTs. The presence of aggregated pores, high surface area, and the electronic structure of CNTs may present a steric effect for compounds and promote efficient oxidation reactions. It was reported that the highest oxidation peak of uric acid occurred in the presence of β-CD with MWCNTs in comparison with the electrode without β-CD which highlighted the role of β-CD in the encapsulation of uric acid [53]. The role of MWCNTs in this electrode was found to reduce the over potential of ascorbic acid and produce a large peak difference between uric and ascorbic acids [53]. β-CD with MWCNTs offered the best platform for the separation of uric acid [53]. In another experiment, electrodes were modified with CNTs in pH 5 phosphate buffer which led to the voltametric resolution of dopamine and ascorbic acid [155]. Collectively, owing to the unique electronic, mechanical, physical, and chemical properties of CNTs and its high aspect ratio, they have the potential to be used to modify electrodes for the resolution of compounds.

Carboxylic-MWCNTs have been used in CZE and have shown promising achiral separations of purine and pyrimidine bases. Liquid chromatography has been widely used for the separation of pyrimidine and purine bases due to its high sensitivity, selectivity and reproducibility [158,159,160]. Capillary electrophoresis also showed promising separation behaviour for the polar and even not fully ionized compounds including pyrimidine and purine bases [161,162]. Capillary electrophoresis also offers savings in time and money, and only requires a small amount of sample [163]. It was reported that simple buffers, such as carbonate or borate, in CZE were able to separate purine and pyrimidine bases but it was difficult to perform baseline separation especially for adenine and thymine [157]. MWCNTs have been modified with carboxylic groups on the surface to enhance their solubility and reactivity and added in the buffer system to investigate the CE separation of six purine and pyrimidine bases, C, A, G, T, 8-azaadenine 8-AA, U, and hypoxanthine HX upon incorporating the carboxylic multi-walled carbon nanotubes [156]. All bases have been separated except T and A in the absence of c-MWCNTs. Adding β-CD to the buffer system and without MWCNTS resulted in partial separation of A and T [156]. Upon incorporation of MWCNTs, with different concentrations to the buffer system in CE, however, there was a gradual improvement in the separation of A and T [156]. Using optimum concentrations of MWCNTs (8.0 × 10^−5^ g/mL), the separation of A and T was achieved [156]. As the concentration of MWCNTs increased, the osmoelectric mobility reduced and thus, the migration time was enhanced [76]. MWCNTs are thought to form a network-like structure which plays an important role in the separation process.

The network formed by MWCNTs may occur as a result of hydrogen bonding and van der Waals forces within their structure [76]. The pores of this network acts as a sieving structure that interacts with some compounds and retain them while allowing others to pass through. Therefore, it was not surprising to find that A eluted after T as the molecular size of adenine is larger than that of thymine. The potential molecular sieving properties of carbon nanotubes and graphene has been recently reported [140,141]. When TX100 0.1 mM was added as a surfactant to keep c-MWCNTs suspended in the buffer system, adenine and thymine eluted at much earlier time compared to c-MWCNT in buffer solution in the absence of TX-100. The role of TX-100 is to maintain the particles of c-MWCNT in suspension which indirectly improves the resolution of A and T [156]. Under optimum conditions of buffer pH, voltage, buffer concentration, c-MWCNTs concentration, the best separation of A and T was observed. Incorporation of c-MWCNTs into a buffer solution is limited to the enhanced achiral separations of adenine and thymine.

Carboxylic-MWCNTs have been also added into ionic liquids coupled with HPLC for use in the separation and determination of thiochromanones in urine. Ionic liquids consist of an organic cation and an inorganic or organic anion and characterise by having high ionic conductivity and high thermal and chemical stability and negligible vapour pressure [164]. They are capable of producing many interactions such as hydrogen bonding, dispersion, ionic exchange, electrostatic and π–π, n–π interactions [165]. They can also increase the stability of nanomaterials. Combining carbon nanotubes with ionic liquids is beneficial since it has been reported to increase the surface area and the possible types of interactions with analytes [166]. Carboxylated MWCNTs were functionalized with amine-terminated ionic liquid and solid phase extraction coupled with HPLC, successfully separated and determined the quantity of thiochromanones in urine [166]. In another experiment, the functionalization of MWCNTs with imidazolium ionic liquid was proved to be useful for the separation of hydroquinone isomers [167]. The results revealed that combining CNTs with ionic liquid not only improves the separation of compounds but also the dispersion of CNTs. Magnetic MWCNTs and ionic liquid have been developed together for the separation of flavonoids in human urine [168]. Developing MWCNTs with an ionic liquid as silanol suppressor with SPE-HPLC system improved the chromatographic separations of antidepressants [169]. The incorporation of MWCNTs into room temperature ionic liquid, thus, is considered an effective way to enhance the separation of compounds in biological samples.

## 7. SWCNTs for Achiral Separation in Purification

Analytical and preparative chromatographic purification methods of compounds are crucial in organic synthesis [170]. The use of HPLC, Ion-Exchange High Performance Liquid Chromatography (IE-HPLC) and Polyacrylamide Gel Electrophoresis (PAGE) have successfully been used as purification methods. For example, purification of peptides by HPLC and CE has been widely reported [170,171]. It is important to find out an easy, efficient and fast method for the separation of impurities including the solvents such as toluene, n-hexane, ethyl acetate and methanol. The use of CNTs allowed the achiral separation of a mixture containing phenol, uracil, *N*, *N*-diethyl-*m*-toluamide and toluene. Aniline compounds are important precursors in the synthesis of many chemicals, and hence the final purification from aniline derivatives is required for the purification of synthesized products. SWCNTs-alginate gel beads were synthesized as a stationary phase in HPLC and showed efficient separation and purification of natural products such as alkaloids. The following two sections discuss the use of CNTs in achiral separation for the chromatographic purification purposes as one of their potential applications.

**Figure 9:**
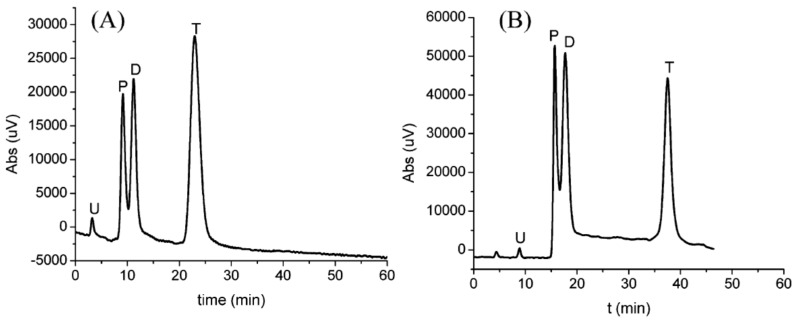
Effect of SWCNTs-coated monolithic column on the separation of a mixture containing phenol (P), toluene (T), uracil (U) and *N*, *N*-diethyl-*m*-toluamide (D) “Reproduced with permission from Y. Li et al. [49]. Copyright American Chemical Society, 2005”. (**A**) Reference column; (**B**) SWCNTs-coated monolithic column.

Monolithic columns have been widely used in CE and HPLC due to their good permeability, ease of in situ preparation and organic and inorganic active surfaces [172]. These advantages have led to researchers using monolithic columns instead of granular packed columns [173]. Thus, there are major advantages in combining SWCNTs and monolithic polymer-based columns. First, it is known that SWCNTs are not soluble in common solvents and hence, well dispersed SWCNTs are required for polymer-based columns. Thus, SWCNTs were activated with hydroxyl groups by treatment with H_2_SO_4_/H_2_O_2_ and hence, the solubility of SWCNTs was improved. Thereafter, the suspension was stabilized by sonication in 2-propanol. Then, the stabilized SWCNTs suspension in 2-propanol acted as a porogen and was added into the polymer mixture of vinyl benzyl chloride, a monomer, and ethylene glycol dimethyacrylate, a cross-linker, to produce a monolithic stationary phase [49]. This polymer mixture incorporating SWCNTs in the monolithic column was found to separate a mixture containing phenol, toluene, uracil and *N*, *N*-diethyl-*m*-toluamide as compared with no separation by the polymer mixture in the absence of SWCNTs [49]. The presence of SWCNT in the polymer mixture was found to increase the retention factors as compared to the polymer mixture without SWCNTs. The increase in the retention times for all compounds may be caused by the possible reduction in the permeability of the polymer mixture due to the addition of SWCNTs. It was proposed that CNTs may alter the pore diameter and distribution which ultimately improved the resolution of the tested compounds. The pore size and properties of the monolithic structure, however, was found to be the same in the presence and absence of SWCNTs [49]. The analytes may be retained in the high surface area network-like channels leading to an increase in retention times (Figure 9) [49].

**Figure 10:**
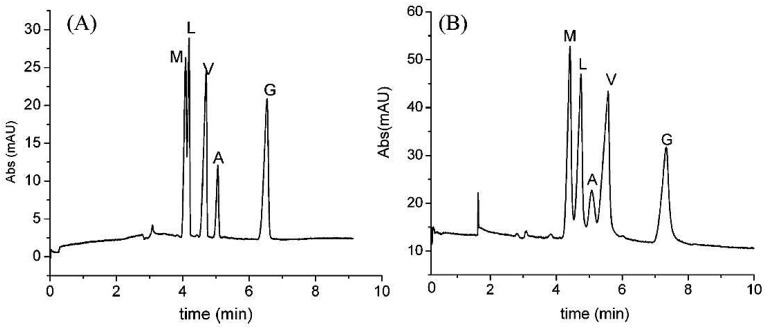
Effect of SWCNTs coated monolithic column on the separation of a peptide mixture “Reproduced with permission from Y. Li et al. [49] Copyright American Chemical Society, 2005”. (**A**) Polymer monolithic column containing SWCNTs; (**B**) Polymer monolithic column in the absence of SWCNTs, Val-Tyr-Val (V), leucine enkephalin (L); (**A**) angiotensin II, methionine enkephalin (M), Gly-Tyr (G).

Incorporation of SWCNTs in the polymer mixture of a monolithic column resulted in a slight change in the pore size and significant increase in the surface area [49]. As well, the incorporation of SWCNTs in the polymer mixture monolithic column was found to increase the retention time and improve the separation of a peptide mixture including angiotensin, Val-Tyr-Val (V), leucine enkephalin (L), II (A), methionine enkephalin (M), Gly-Tyr (G) (Figure 10) [49]. The order of elution of these peptides by the monolith column containing SWCNTs was different from the column that did not contain carbon nanotubes (Figure 10) [49]. Collectively, CNTs in a polymer-based monolithic column were found to alter the retention times and improve the separation of some compounds and peptides in micro-HPLC and CEC [49].

In another experiment, SWCNTs have been combined with poly diallyldimethylammonium chloride (PDDA), a positively charged polymer and were coated to the interior surface of a capillary column and showed achiral separation of some basic proteins when used for capillary electrophoresis [174]. PDDA was used to coat the inside surface of a fused capillary that made it suitable for the physical attachment of c-SWCNTs [50]. CNTs are hydrophobic and insoluble in most solvents and thus, carboxylation of its outside surface occurred. Although CNTs are hydrophobic in nature and suitable for the adsorption of lipophilic analytes, they have ionic interactions with many molecules if they are functionalized with –COOH. For example, unmodified CNTs were unable to interact with most metals due to their high hydrophobicity [175], while these metals can be adsorbed on the surface of the modified CNTs [176]. Since CNTs are hydrophobic in nature, they strongly interacted with the ‘1-pyrenebutanoic acid succinimidyl ester’ through π-stacking [177]. Seven protonated aniline derivatives were selected to investigate their separation using c-SWCNTs-PDDA under an electrical field [50]. Since the pKa of these compounds are similar, baseline separation, in a buffer solution at pH 5.5 to 9 and in the absence of c-SWCNTs-PDDA, was not observed [50]. The control column was only able to partially separate these aniline derivatives where 2- and 3-chloroaniline overlapped [50]. There was no baseline separation of aniline from *o*-aniline when the reference column was used [50].

**Figure 11:**
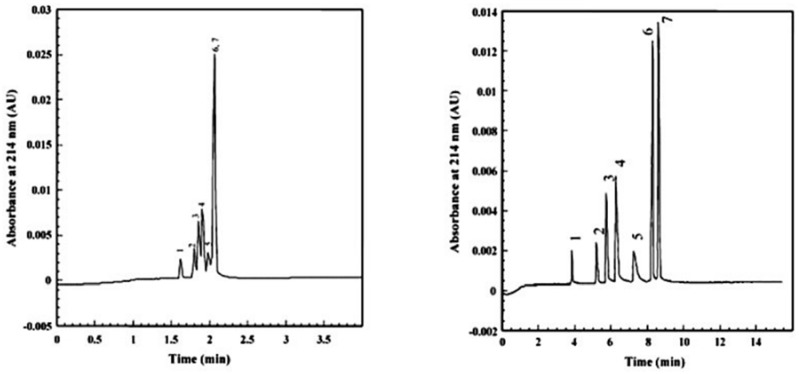
Effect of SWCNTs-PDDA coated capillary in CE on the resolution of a mixture containing seven aniline derivatives “Reproduced with permission from [50]. Copyright Elsevier, 2005”. (**A**) A bare fused capillary; (**B**) SWCNTs-PDDA-coated capillary; (1) 3-aminophenol (3-AP), (2) aniline, (3) *o*-anisidine, (4) 4-aminophenol (4-AP), (5) 4-chloroaniline (4-ClA), (6) 3-chloroaniline (3-ClA) and (7) 2-chloroaniline (2-ClA).

When a PDDA coated capillary was used, with the voltage ranged from +20 kV to 30 kV, there was no separation [50]. Baseline separation of all aniline derivatives, however, was achieved by using a c-SWCNTs-PDDA column even under the maximum voltage +30 kV (Figure 11) [50]. Baseline separation in the presence of c-SWCNTs could be attributed to the hydrophobic nature of their six-membered ring carbon structure and the ionic nature of its –COOH that allowed adsorption of both lipophilic and ionic analytes [50]. It has been reported that c-SWCNTs bind with PDDA via π–π stacking interactions [178]. In addition, CNTs are thought to form π-π stacking interactions with benzyl groups of the aniline derivatives [50]. Ionic interactions can also occur between the protonated aniline analytes and the carboxylic SWCNTs [50]. In brief, due to their high surface area, hydrophobic and ionic sites of interaction, CNTs are considered promising candidates for the separations in CE and chromatography [50].

## 8. Separation of Isomers of CNTs

Extensive efforts have been made in order to separate carbon nanotubes with certain chirality [179]. The separation of metallic and semiconducting SWCNTs has been achieved [180]. Such chromatographic separation was based on their chirality and/or diameter and length [181]. There have not been enough studies on the separation of the optical isomers (right or left-handed) of the chiral CNTs [182,183]. A mixture containing equal amount of the isomers of CNTs is unable to rotate the polarized light as each isomer cancels the effect of the other. A new technique was used to enrich both of the optically active isomers of carbon nanotubes which was based on using chiral nano-tweezers to separate the right and left handed isomer of CNT with discrete chirality. Unlike other methods that use DNA or a surfactant [96], this technique is simple and results in pure optically active CNTs without impurities from the tweezers [179]. The (*S*) or (*R*)-nano-tweezer was used to extract the optically active isomers of SWCNTs including different chiral indices such as (7, 5), (8, 4), (6, 5) and (8, 3). At first, SWCNTs were sonicated with the nano-tweezer dipophyrin and then centrifuged [179]. Thereafter, the optically active forms of CNTs were extracted from the tweezer and solubilized in D_2_O and sodium dodecylbenzenesulfonate (SDBS) [179]. The nano-tweezers were then completely removed by washing with pyridine and THF solvents. As a result, the mirror image of all tested indices (7, 6), (7, 5), (8, 3), (8, 4) and (6, 5) were obtained [179]. This method provides researchers with the opportunity to obtain the pure optically active form of carbon nanotubes that can be used for the enantioseparation of pharmaceuticals.

## 9. Advantages and Drawbacks

CNTs are characterized with unique physical, mechanical and chemical properties that render them suitable for nanotechnological and chromatographic applications [184]. They possess high surface areas and their length is more than any other materials and thus, they act as large platforms for the interactions with the analytes [184]. They are stable at high temperatures; therefore, they function well in both extremely cold and hot conditions [184]. They can be easily modified with other chiral selectors and therefore, can be used to enhance enantioseparations [58,59,60,185,186]. Furthermore, SWCNTs as well as MWCNTs possess chirality in their structures and they have shown direct enantioseparation in some studies [33,47,59]. CNTs played an important role in chiral and achiral separations in pharmaceutical, biological, environmental and medical studies [33].

CNTs are, however, relatively new materials and their production and purification are still costly [187]. CNTs are highly hydrophobic which hinders their abilities to be dissolved in many organic and aqueous solvents [188,189]. Although they possess chirality in their structure, this can be impeded if they are not pure [11]. Although CNTs are chiral in their structure, they are produced as a racemic mixture which indicates the importance of obtaining the pure optically active forms [11]. Some studies indicated that CNTs can perform direct and indirect enantioseapration as CNT racemic mixtures are not produced in equal proportions. Functionalization of CNTs with carboxylic groups and sonication can also be another factor that reveals the chirality of CNTs [190,191]. However, functionalized CNTs are less thermostable than non-functionalized CNTs [192,193]. Thus, it is advisable to keep the temperature below 140 °C to avoid losing the functionalization of CNTs [192,193]. There is still no solid explanation of how CNTs can directly undergo chiral separation. 

## 10. Future Perspectives

CNTs have emerged as one of the most useful materials in a wide range of applications including nanotechnology and chromatography. It is important to separate and purify carbon nanotubes with discrete chiral characteristics for the application in separation science. CNTs have been widely applied to facilitate the enantioseparation by their incorporation in a monolithic columns or immobilization of a chiral selector on their surfaces. Although functionalization of CNTs with carboxylic, amino, bromo, hydroxyl groups have been widely reported, further studies are recommended to immobilize a wide range of chiral selectors on CNTs to extend their applications in chiral and achiral separations. Magnetization of CNTs is one of the fastest, easiest and cheapest ways for batch solid-phase extraction. Although modification of electrodes with CNTs has been also applied for voltametric separation of few compounds, this technique can be useful for the chemical separation of other structurally-related compounds. Direct enantioseparation by CNTs, however, have been reported by few researchers. Chiral CNTs exist in the racemic form and thus, it is significantly important to exert more research on the separation of pure and optically active CNTs which are expected not only to facilitate the interaction with the analytes but also directly separate a wide range of compounds, probably better than many existing chiral selectors. The exact mechanism of separation by CNTs is still unclear and untouched in this article. Different prospects based on the three point interaction have been proposed to explain the ability of CNTs to adsorb the analyte to their surfaces. The application of CNTs in separation science is still in the infancy. More research is needed to enhance the chirality and enantioseparation by CNTs and to reach the potential mechanism of separation.

## Figures and Tables

**Table 1:** Different approaches for the enantioseparation with CNTs.

Template	Format	Analyte	Analysis	Reference
β-CD-MWCNTs	Pseudo-stationary phase	Clenbuterol	CE	[48]
HP-β-CD-MWCNTs	Added in stationary phase	Clenbuterol	TLC	[59]
BSA-SWCNTs	Stationary phase	Tryptophan	MCE	[60]
Chiral ion liquid-SWCNTs	Chemical bonding	Amino acids, carvone, (*dl*) leucine, of (±)-*N*-phenyl-α-methylbenzylamine	GC	[37]
SDS-MWCNTs	Pseudo-stationary phase (partial filling)	Ephedrine and norephedrine	EKC	[32]
*l*-Threonine-MMWCNTS	Modification with chiral selector	(*dl*) Mandelic acid	Magnetic field	[51]
SWCNTs-polymer based column	Encapsulation in monolithic column	Etozoline, celiprolol, cizolirtine, miconazole, sulconazole, nomifensine, chlorpheniramine	Nano-HPLC	[46]
PNA-CNTs	Immobilization on CNTs coated monolithic column	Ten amino acids	HPLC	[47]

**Table 2:** Different approaches for achiral separation by CNTs.

Template	Format	Analyte	Analysis	Reference
SWCNT	Added in monolithic polymer based column	Phenol, toluene, uracil and *N*, *N*-diethyl-*m*-toluamide	HPLC	[49]
MWCNTs	Modified with electrodes	Uric acid and ascorbic acid or dopamine and ascorbic acid	Voltametric separation via electrodes	[53,136]
Carboxylic SWCNTs	Added in run buffer	Theobromine, caffeine or epinephrine and *dl*-noradrenaline or catechol and hydroquinone	CE	[76]
c-MWCNTs	Added in run buffer	Six pyrimidine and purine bases	CZE	[137]
SWCNTs-PDDA	SWCNTs encapsulated in fused capillary coated with PDDA	Seven aniline derivatives	CE	[50]
MMWCNTs	Magnetization of MWCNTs with iron oxide nanoparticles	Lead and manganese	Magnetic field	[52]
